# Toxigenic *Corynebacterium diphtheriae* Infections in Low-Risk Patients, Switzerland, 2023

**DOI:** 10.3201/eid3101.241138

**Published:** 2025-01

**Authors:** Pascal Urwyler, Daniel Goldenberger, Kerstin Grosheintz, Rahel Tarnutzer, Maike Markstein, Celine Sucker, Anna-Maria Balestra, Lukas Merki, Michelle Baumann, Nicolas Gürtler, Aurélien Emmanuel Martinez, Matthias von Rotz, Branislav Ivan, Claudia Lang, Pascal Schläepfer, Peter M. Keller, Eva Wuerfel, Sarah Tschudin-Sutter

**Affiliations:** University Hospital Basel, University of Basel, Basel, Switzerland (P. Urwyler, D. Goldenberger, M. Baumann, N. Guertler, A.E. Martinez, M. von Rotz, B. Ivan, C. Lang, P. Schläepfer, P.M. Keller, S. Tschudin-Sutter); Canton of Basel-Stadt, Basel (K. Grosheintz, R. Tarnutzer, M. Markstein, C. Sucker, E. Wuerfel); St. Claraspital Basel (A.-M. Balestra, L. Merki)

**Keywords:** *Corynebacterium diphtheriae*, bacteria, Switzerland, diphtheria, outbreak investigation, public health

## Abstract

We report a cluster of infections with genetically related toxigenic *Corynebacterium diphtheriae* linked to an outbreak among asylum seekers in Switzerland that subsequently affected patients without known exposure. This discovery highlights the importance of rapid, interdisciplinary outbreak investigations and regular vaccination status assessment, especially in elderly populations with waning immunity.

Toxigenic *Corynebacterium diphtheriae* has reemerged in recent years and has been linked to several outbreaks worldwide. Most outbreaks occurred in low-resource settings, and mortality rates have ranged from 0.5%–0.8% in the Rohingya population in Bangladesh to 42.9% in infants in Nigeria (*1*,*2*). Higher mortality rates have been associated with poor vaccine coverage and nonavailability of antitoxin (*3*). Since June 2022, disease surveillance authorities in Europe have reported an increase in diphtheria cases, linked mainly to refugees from Syria and Afghanistan (*4*). Most centers reported primarily cutaneous cases, but 2 *C. diphtheriae*–related deaths from respiratory diphtheria occurred in Austria and Belgium (*5*,*6*).

In Basel, Switzerland, a cluster of diphtheria infections occurred at a national asylum center in August 2022 (*7*). After testing, contact precautions, vaccination, and antimicrobial treatment and prophylaxis were implemented, the outbreak was controlled (*8*). Whole-genome sequencing (WGS) revealed the presence of 3 different sequence types (STs), 377, 384 and 574, with relatedness identified among only some of the clinical isolates, suggesting that most were imported to the asylum center. Other national asylum centers in Switzerland and Europe reported similar case clusters of *C. diphtheriae* infection; some isolates were macrolide-resistant (*9*).

## The Study

In October 2023, healthcare workers diagnosed 3 clinical cases of diphtheria within 7 days at the University Hospital Basel and the St. Claraspital Basel (Basel, Switzerland). One patient with cutaneous diphtheria was homeless; the other 2 patients had no obvious risk factors. One of the 3 patients demonstrated signs of toxin-mediated disease.

Patient A, a 40-year-old homeless man, arrived at the hospital with painful, encrusted lesions on his head and forearm. Topical treatment resulted in improvement of the lesions, leading to the patient’s discharge. After his discharge, swab samples of the lesions grew toxigenic *C. diphtheriae*. Ten days later, the patient returned to the hospital and received antimicrobial treatment (Appendix Table). Pharyngeal swab samples for *C. diphtheriae* remained negative. The patient’s vaccination status was not known.

Patient B, a 78-year-old woman, visited the hospital with acute respiratory failure related to suspected pneumonia and underlying chronic obstructive pulmonary disease. Hospital staff initiated antimicrobial drug treatment and noninvasive ventilation. Bronchoscopy showed abundant viscous mucus. The patient deteriorated rapidly and died within 24 hours. Laboratory investigations revealed *C. albicans*, interpreted as a colonizer, and toxigenic *C. diphtheriae*, cultured postmortem from the patient’s bronchial aspirate on nonselective media. Despite the absence of pseudomembranes, the clinical findings indicated toxin-mediated *C. diphtheriae* infection. Growth of *C. diphtheriae* in 1 of 2 blood cultures after 92 hours supported this suspected diagnosis. The patient’s vaccination status was not known. Antitoxin treatment was not administered because the patient died before laboratory findings were available.

Patient C, an 88-year-old man, visited the dermatology outpatient clinic for a chronic sacral ulcer. He transitioned to hospital care after a local biopsy revealed growth of toxigenic *C. diphtheriae.* Subsequent pharyngeal swab samples remained negative. The patient improved after treatment with systemic antibiotics and local wound care. He reported receiving no diphtheria vaccine since one he received in 1969. His diphtheria antitoxin IgG level was <0.1 IU/mL, indicating waning immunity.

Patient A and C showed improved cutaneous lesions at time of discharge, and repeat swab samples remained negative, confirming clearance of *C. diphtheriae*. After identifying *C. diphtheriae*, we cultured wound and pharyngeal swab samples on selective media (tellurite agar) and identified suspected colonies by using matrix-assisted laser desorption/ionization time-of-flight mass spectrometry. We conducted *C. diphtheriae* toxin gene testing by using an in-house, real-time PCR (*10*).

We performed antimicrobial susceptibility testing, DNA extraction, and sequencing (Appendix). Laboratory diagnosis, including PCR, is performed decentrally in Switzerland, and WGS is not performed routinely on a national level. Diphtheria diagnoses require notification to the Federal Office of Public Health. WGS is regularly conducted at our institution to analyze rare or multidrug-resistant bacteria as part of routine surveillance. Sequencing of the isolate of *C. diphtheriae* from patient A was not successful, but the 2 clinical isolates from patient B and C revealed genetically identical (zero differences in single-nucleotide polymorphisms) strains of ST574. Both isolates were closely related (6–16 differences in single-nucleotide polymorphisms) to a cluster of strains derived from 5 patients accommodated in a federal asylum center in Basel, Switzerland, in 2022 (Figure). 

**Figure:**
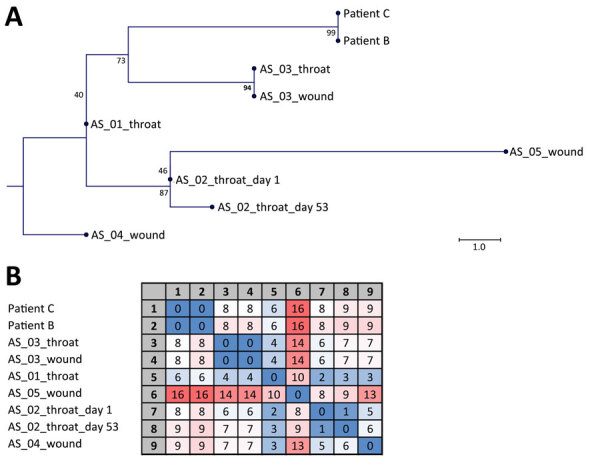
Sequencing data from study of toxigenic *Corynebacterium diphtheriae* infections in low-risk patients, Switzerland, 2023. Maximum-likelihood single-nucleotide polymorphism (SNP) phylogeny (A) and SNP matrix (B) of *C. diphtheriae* isolates derived from patient B, patient C, and 5 asylum seekers (AS). Numbers at the tree nodes in panel A denote bootstrap values as percentages from 1,000 replicates; scale bar indicates substitutions per site. Matrix in panel B contains the pairwise number of SNP differences used to generate the tree. We used a 3-color scale, where blue represents lowest values, red highest values, and white intermediate values.

Patient A was linked only temporally to the 2 other cases. However, the genetic relatedness of the other strains prompted a joint outbreak investigation that involved public health authorities. Patients B and C had no epidemiologic link to each other or to previously diagnosed cases and reported no travel history or contact to other persons with compatible symptoms or considered at risk. Patients B and C received home care; however, no careworker was involved in the care of both patients. We determined no evident link between the home caregivers and the previous cases from 2022. We obtained respiratory swabs from 3 nurses involved in home care and tested samples for *C. diphtheriae*; all tests were negative. Respiratory swab samples of 14 persons considered to be close contacts of patients B and C were also negative. We recommended postexposure chemoprophylaxis and vaccinations (if the last diphtheria vaccination was >5 years previously) to all contacts. The cantonal Department of Health initiated an awareness campaign underlining the importance of vaccination regimens. Regional authorities subsequently initiated a national dialogue regarding migration health.

The close genetic relatedness of 2 strains of the *C. diphtheriae* in our study to previously isolated strains indicated a reintroduction of toxigenic strains to Europe by migration. Both patients with sequencing results had infections with isolates of ST574, the ST most commonly reported in Switzerland during an outbreak in 2022 (A. Hoefer et al., unpub. data).

We believe that ensuring early diagnosis of *C. diphtheriae* infections relies greatly on improving awareness among healthcare and laboratory personnel. In view of increasing antimicrobial resistance, susceptibility testing should be performed to ensure successful treatment. Confirmatory laboratory tests and antitoxin should be readily available, and healthcare workers should consider antibiotic postexposure prophylaxis for contact persons at risk.

The severity of disease in the cases we studied, leading to death in 1 patient, might indicate waning immunity and lack of catch-up vaccinations, particularly in the elderly population. One report estimated full seroprotection against diphtheria to be 50% in the general population in England (*11*). Although the immunization status of patients A and B remained unclear, patient C had low antitoxin IgG levels, underscoring the need for regular review of vaccination status and respective booster vaccinations to protect against toxin-mediated disease. We acknowledge limitations to our investigation of these patients, including lacking WGS data for patient A, missing serologic results for patients A and B, and the absence of Elek testing for all isolates.

Improving vaccination coverage among refugees remains challenging. Migrant populations are especially prone to receiving inadequate medical care. A recent meta-analysis reported a pooled vaccine-coverage for diphtheria of ≈57% in migrant populations in Europe (*12*). Drawing from insights gained in Belgium (*6*), we believe that prioritizing access to comprehensive healthcare services for persons residing in challenging circumstances is a critical preventative measure, not only to safeguard the well-being of these populations but also to uphold public health standards.

## Conclusions

*C. diphtheriae* has emerged in populations usually not considered at risk. We advocate for increased awareness among clinicians, public health authorities, and laboratory personnel, as well as healthcare structures enabling rapid, interdisciplinary outbreak investigation. Furthermore, given that *C. diphtheriae* infections can lead to potentially fatal outcomes, we recommend a more comprehensive approach to assessing vaccination status, especially in elderly populations but even in segments of the population with no evident risk for exposure.

Appendix Additional information for toxigenic *Corynebacterium diphtheriae* infections in low-risk patients, Switzerland, 2023.
