# Supplementary material for: Toxigenic Corynebacterium diphtheriae Infections in Low-Risk Patients, Switzerland, 2023
Source: Emerg Infect Dis. 2025 Jan;31(1):164–7. doi: 10.3201/eid3101.241138 (PMC11682798; doi:10.3201/eid3101.241138)
Supplement: Appendix — Additional information for toxigenic Corynebacterium diphtheriae infections in low-risk patients, Switzerland, 2023. [file 24-1138-Techapp-s1.pdf]

Article DOI: http://doi.org/10.3201/eid3101.241138

EID cannot ensure accessibility for supplementary materials supplied by authors. Readers who have difficulty accessing supplementary content should contact the authors for assistance.

Toxigenic *Corynebacterium diphtheriae* Infections in Low-Risk Patients, Switzerland, 2023

Appendix

Antimicrobial Susceptibility Testing and Antibiotic Treatment

Antimicrobial susceptibility testing was performed using gradient diffusion test strips and interpreted according to the European Committee on Antimicrobial Susceptability Testing (EUCAST) version 13 (https://www.eucast.org/clinical_breakpoints) for penicillin and Clinical & Laboratory Standards Institute (CLSI) criteria (https://clsi.org/standards/products/microbiology/documents/m45) for erythromycin. Isolates from all 3 patients showed intermediate susceptibility to penicillin with minimal inhibitory concentrations (MICs) of 0.125–0.38 mg/L and were tested fully susceptible to erythromycin with MICs of 0.047–0.064 mg/L. Elek-testing is not routinely performed at our institution, so that we cannot exclude that the organisms were “non-toxigenic tox gene-bearing” isolates.

DNA Extraction and Sequencing

DNA is extracted using a EZ1 DNA Tissue Kit in combination with EZ1 Advanced Instrument (Qiagen, Hilden, Germany). Sequencing libraries are built using Nextera DNA flex Library Prep followed by sequencing using NextSeq500 by Illumina. CLC Genomics Workbench Version 23.0.5 was used to create a custom workflow (Workflow_QC_trim_map_variant_101070_CLC23_0_5; .clc file available from corresponding author) where reads were trimmed and mapped to a reference (NCBI RefSeq assembly GCF_001457455.1). A phylogenetic tree based on single nucleotide polymorphism variants was called, producing a distance matrix and maximum likelihood tree.

Appendix Table. Antibiotic treatment of all 3 patients in a study of toxigenic *Corynebacterium diphtheriae* infections in low-risk patients, Switzerland, 2022

Patient	Antibiotic treatment	Treatment duration, d
Patient A	Amoxicillin	2
	Amoxicillin-clavulanic acid	8
	Azithromycin	3
Patient B	Amoxcillin-clavulanic acid plus clarithromycin	2
Patient C	Amoxicillin	2
	Amoxcillin-clavulanic acid	6
	Amoxicillin	8
